# Mitigating the risk of tube shortages: A blood collection tube validation study conducted in South Africa

**DOI:** 10.4102/ajlm.v14i1.2628

**Published:** 2025-04-23

**Authors:** Marizna Korf, Jody Rusch, Aye Aye Khine, Nalene Strauss, Lourens Jacobsz, Annalise E. Zemlin, Helena Vreede

**Affiliations:** 1 Division of Chemical Pathology, Faculty of Health Sciences, Stellenbosch University, Cape Town, South Africa; 2 Division of Chemical Pathology, National Health Laboratory Service, Tygerberg Hospital, Cape Town, South Africa; 3 Division of Chemical Pathology, Faculty of Health Sciences, University of Cape Town, Cape Town, South Africa; 4 Division of Chemical Pathology, National Health Laboratory Service, Groote Schuur Hospital, Cape Town, South Africa; 5 Division of Chemical Pathology, National Health Laboratory Service, Green Point Laboratory, Cape Town, South Africa

**Keywords:** blood specimen collections, pre-analytical phase, risk management, validation study, South Africa

## Abstract

**Background:**

The National Health Laboratory Service was using Becton Dickinson (BD) blood drawing tubes and, in 2021, the supplier notified customers of supply challenges, indicating a risk of global shortages.

**Objective:**

This study aimed to validate candidate blood collection tubes from four brands (VACUCARE, VACUETTE^®^, VACUTEST^®^, and V-TUBE^™^) compared to BD Vacutainer^®^ tubes in three National Health Laboratory Service laboratories in Cape Town, South Africa.

**Methods:**

Blood was collected from 300 healthy volunteers between October 2021 and November 2021. The technical validation assessed 11 quality indicators, with a sigma metric greater than 4 deemed acceptable. Usability feedback was gathered from phlebotomists. The clinical validation estimated differences in results across 52 clinical chemistry tests, using desirable bias specified by the European Federation of Clinical Chemistry and Laboratory Medicine Biological Variation Database, or Ricos, as acceptance criteria. Analysis was performed on Roche cobas^®^ 6000 and DiaSorin Liaison^®^ XL analysers.

**Results:**

All VACUCARE tubes exhibited sigma metrics above 4, indicating excellent performance. VACUETTE^®^ and V-TUBE^™^ were not uncapped by all Roche pre-analytical systems. VACUTEST^®^ caps had rigid rubber, making it more challenging to puncture and detach the tube, which resulted in needle displacement. Both VACUCARE and V-TUBE^™^ were reported as user-friendly. All candidate tube analytes showed acceptable clinical performance.

**Conclusion:**

VACUCARE, VACUETTE^®^, VACUTEST^®^ and V-TUBE^™^ are viable alternatives to BD Vacutainer^®^. However, based on the results obtained from the technical validation, VACUCARE was identified as the most suitable interim replacement for BD Vacutainer^®^ during the shortage.

**What this study adds:**

This study addresses a gap in the literature on tube validation and provides valuable insights for clinical laboratories considering a replacement. It also presents an alternative approach to technical validation by utilising sigma metrics.

## Introduction

Blood collection tube technology has advanced, with the use of complex materials and additives designed to enhance convenience and improve turnaround time. These tubes are an important, and often under-recognised, pre-analytical consideration in clinical laboratories, since these devices are used to analyse patient samples on complex state-of-the-art equipment. The tube type, its additives, and analyte stability under various storage conditions, can potentially contribute to significant error. Results of laboratory tests are critical to patient care, and precision and accuracy are essential. Manufacturers and clinical laboratories should, therefore, validate the technical and clinical performance of new blood collection products.^1,2^ Proper tube validation is crucial to prevent the financial and human consequences related to inaccurate testing. These include unnecessary testing, delayed results, and compromised patient care.

Several factors, including supply chain disruptions driven by transportation delays, constrained supplies, and increased costs, as well as the increased demand during the Coronavirus disease 2019 pandemic, imposed unprecedented challenges on the entire healthcare industry. As a result, Becton, Dickinson and Company (BD, UK Limited, Berkshire, United Kingdom), a prominent manufacturer of blood collection tubes, notified customers of supply challenges with the risk of global product shortages in 2021.^3^

Following this notice, the National Health Laboratory Service of South Africa, which is mandated by government to provide clinical laboratory services to over 80% of the population, sent a notice to healthcare workers with guidance on the efficient use of blood collection tubes. Clinicians and phlebotomists were urged to refrain from collecting spare tubes, since a properly filled blood collection tube will be sufficient for all tests requested, in most cases. Clinicians were reminded to first review patients’ results before ordering more tests, to avoid unnecessary testing and re-testing, and add-on requests were encouraged. Additionally, legible completion of request forms and good phlebotomy technique were advised to prevent unnecessary rejections. Although the current blood tube shortage has been resolved, these measures should continue to be practised, promoting environmental stewardship and sustainable laboratory practices.

To mitigate tube shortage risk, the research team conducted a comprehensive blood collection tube validation study. This was considered a validation study in accordance with the International Organization for Standardization 9000:2005 specifications, which define validation as the ‘confirmation, through the provision of objective evidence … that the requirements … for a specific intended use or application have been fulfilled’.^4^ Additionally, the terminology aligns with that used by the European Federation of Clinical Chemistry and Laboratory Medicine (EFLM) Working Group for Pre-Analytical Phase in their opinion paper on validating blood collection tubes in clinical laboratories.^5^ While the validation protocol followed EFLM Working Group for Pre-Analytical Phase guidance, the study’s unique circumstances involved a global tube shortage. Unlike the usual transitions to new tubes following the awarding of a new tender, the urgent aim was to identify and validate alternative options to mitigate potential supply risks for the National Health Laboratory Service. Blood collection tubes from four brands (VACUCARE, VACUETTE^®^, VACUTEST^®^, and V-TUBE^™^ [Table 1]) were validated to identify the closest match to the BD tubes’ performance, with the intention of returning to BD tubes once supply issues were resolved.

**TABLE 1:** Manufacturer details and lot numbers of blood collection tubes used during the study at all three testing sites in Cape Town, South Africa, October 2021 to November 2021.

Type types	SS	K_2_EDTA	LiH†	NaFl/KOx
Tube volume	5 mL	4 mL	4 mL	4 mL
**VACUCARE**	210314	210505	200618	210611
Zhejiang GongDong Medical Technology Co., Ltd, Taizhou, China‡		210608		
**VACUETTE^®^**	A21053HC	A21013NY	A21083T4	A20093X5
Greiner Bio-One GmbH, Kremsmünster, Austria	A201049T			
**VACUTEST^®^**	KZ1801	WZ1321	KZ2561	KZ1521
VACUTEST Kima SRL, Padua, Italy	KG1371	WZ2501		
**V-TUBE™**	6319036	2519002	27110023	Tube not available
AB Medical Inc, Jangseong, South Korea				
**BD Vacutainer^®^**	1064851§	1053105	1099594	1040024
Becton Dickinson and Company, UK Ltd, Berkshire, United Kingdom	1117687§	1116117
	1151158§	1124196		

SS, serum tube with gel separator; K_2_EDTA, dipotassium ethylenediaminetetraacetic acid; LiH, lithium heparin; NaFl/KOx, sodium fluoride/potassium oxalate.

† , technical validation only,

‡ , branded VACUCARE for EREZlabmed, Gauteng, South Africa, trademark pending,

§ , SST^™^ II Advance Plus.

## Methods

### Ethical considerations

The study received full approval from the Health Research Ethics Committee of Stellenbosch University (reference no.: N21/09/095), Cape Town, South Africa, on 12 November 2021. The study was conducted in compliance with the 2013 World Medical Association Declaration of Helsinki. Written informed consent was obtained from all volunteers. Each participant was assigned a unique identifier number during blood collection, which was used for labelling the tubes, registering the samples on the Laboratory Information System, and during data analysis. Only the principal investigator held the key to reidentify participants, allowing the research team to share their blood results with them.

### Participants

**FIGURE 1:**
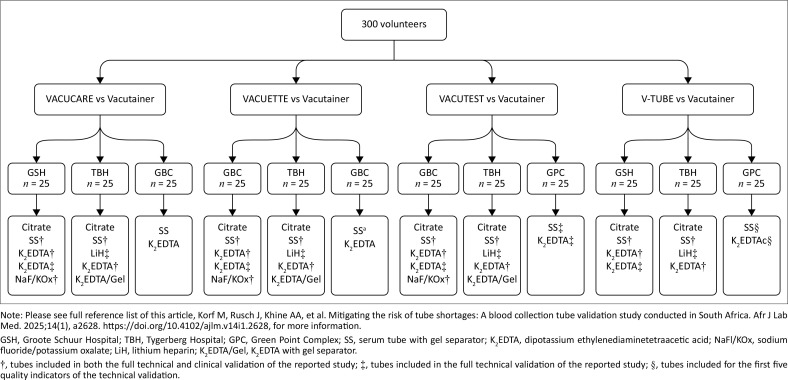
This flow diagram illustrates the participation of volunteers across three study sites in Cape Town, South Africa, from October 2021 to November 2021. The blocks highlighted in grey indicate tubes collected by the same phlebotomy team. Only tubes marked with^†^ or^‡^ were included in the reported study. The other tubes were used for haematology^6^ and therapeutic drug monitoring validations, which are not reported in the current study.

A total of 300 healthy male and female adult volunteers participated in this blood collection tube validation study. The study was conducted across three laboratories in Cape Town, South Africa, from October 2021 to November 2021 (Figure 1).^6^ Investigators sent recruitment invitations to division heads, who then forwarded them to National Health Laboratory Service staff and healthcare workers. Additional participants, including friends and relatives of staff members, joined the study through word-of-mouth awareness. The number of 300 volunteers was selected to ensure an adequate number of sample pairs for the clinical validation and to limit the number of tubes collected per participant to a maximum of five tubes each for candidate and comparative tubes (Figure 1). Exclusion criteria: anaemia or receiving treatment for anaemia or iron deficiency, autoimmune diseases, hereditary haematological disorders, bleeding disorders or anticoagulation treatment, heart conditions with low cardiac output, confirmed or possible pregnancy, and being unwell at the time of the study. The exclusion criteria were included in the recruitment invitations, allowing volunteers to self-screen and only enrol if they met the eligibility requirements.

### Study design

The design of the study was based on the 2016 opinion paper by the EFLM Working Group for Pre-Analytical Phase and the Clinical and Laboratory Standards Institute GP34A guideline.^1,5^ Technical and clinical validations were conducted. ‘Comparative tubes’ refer to the tubes currently used in the laboratory (BD), while ‘candidate tubes’ are those under evaluation as alternatives. Details of comparative and candidate tube manufacturers are summarised in Table 1. The V-TUBE^™^ glucose tube was excluded as it contained ethylenediaminetetraacetic acid, which was incompatible with certain glucose methods used in the National Health Laboratory Service. Lithium heparin tubes were only included in the technical validation, as Troponin T was the only test performed on lithium heparin plasma, and its results were expected to fall below the measuring range within the healthy study population.

### Sample collection

Venous blood samples were collected by experienced phlebotomists using a blood collection device (evacuated tube holder) and 21-gauge needle (or 22-gauge butterfly needle) following Clinical and Laboratory Standards Institute GP41 guidelines.^7^ The same team performed phlebotomy for most of the study, in close proximity to the laboratories (Figure 1). Beckton Dickinson devices were used for both comparative and candidate tubes. Phlebotomy procedures were standardised bilaterally. Comparative tubes were filled from one arm, and candidate tubes from the other, with consistent sides but alternating order. Blood was drawn first from the arm that the phlebotomist assessed as having more challenging venous access, based on their evaluation of vein characteristics such as size, depth, or visibility. This approach helped determine the appropriate device and the number of tubes that could be collected. Tube combinations varied across sites, adhering to the specified draw order. Samples were processed per manufacturers’ instructions and tested immediately or stored under conditions that ensured analyte stability. All analyses were completed within 3 days of collection.

### Technical validation

**FIGURE 2:**
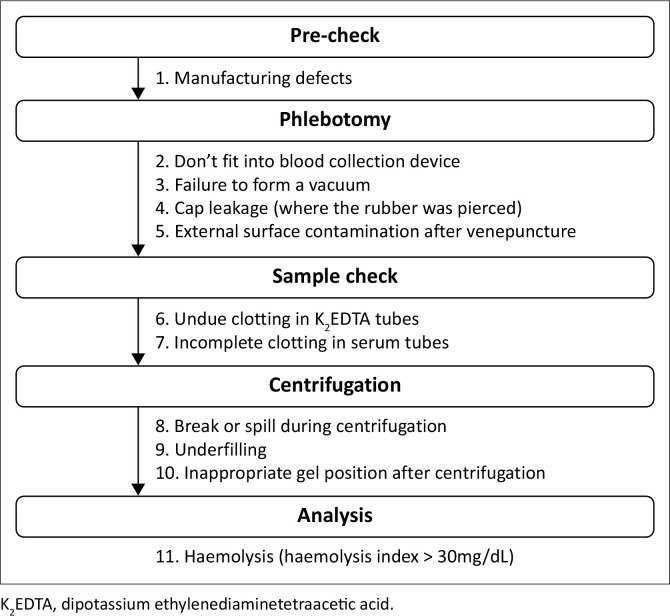
Process flow of technical validation of blood collection tubes with quality indicators checked at each stage – Cape Town, South Africa, October 2021 to November 2021.

Pathology registrars and experienced laboratory technologists assessed 11 quality indicators (QIs) during various study stages (Figure 2). During the pre-check, pack and blood collection tube details, including lot number, expiry date, draw volume, and fill line presence were reviewed. The ‘filling distance’, measured as the distance between the top of the fill line and the bottom of the tube, was used to identify underfilling, defined as less than 90% of the filling distance. Each tube pack was assigned a reference number recorded during phlebotomy.

While the Clinical and Laboratory Standards Institute GP34A guideline recommends visual haemolysis assessment, this method is unreliable.^8,9,10^ Haemolysis was instead measured automatically using Roche analysers (Roche Diagnostics GmbH, Mannheim, Germany), which determine the haemolysis index through absorbance measurements of diluted samples at 570 nm and 600 nm.^11^ To improve accuracy, a quantitative haemolysis index of 30 mg/dL or lower was considered acceptable. This threshold was chosen because several routine chemistry analytes, including potassium, lactate dehydrogenase, and conjugated bilirubin, begin to be affected around this level.^12,13,14^

Tubes showing underfilling or *in vitro* haemolysis during the technical validation were excluded if phlebotomy difficulties were indicated on the phlebotomy checklist. After QI evaluation, the compatibility of all candidate tubes with the pre-analytical systems at the testing sites, namely the Modular Pre-analytics system (Roche Diagnostics GmbH, Mannheim, Germany) and *Probenverteiltechnik* cobas® p 612 pre-analytical system (Roche Diagnostics GmbH, Mannheim, Germany), was assessed. Phlebotomists provided feedback on their user experience.

The global tube shortage limited the study from including the recommended 120 candidate and comparative tubes for the technical validation.^5^

### Clinical validation

The clinical validation aimed to determine whether the tube introduced bias, increasing total error and potentially having a clinically significant effect on patient results.

The Clinical and Laboratory Standards Institute EP09 guideline recommends 100 sample pairs for validation and 40 for verification.^15^ However, because of the tube shortage and other resource constraints, results from routine tests conducted at both Groote Schuur Hospital and Tygerberg Hospital were combined, yielding a total of 50 sample pairs. Additionally, 25 sample pairs from selected routine tests and specialised tests conducted only at a single study site were included.

The choice of analytes was limited by the healthy study population recruited. Analytes expected to have values below the measuring range in healthy participants, such as direct bilirubin, C-reactive protein, free prostate specific antigen, growth hormone, progesterone, and oestradiol, were excluded. In addition, lactate dehydrogenase and aspartate aminotransferase are affected by minor degrees of haemolysis and were also excluded.

Thirty analytes were measured on cobas^®^ c501 (Roche Diagnostics GmbH, Mannheim, Germany) analysers: sodium, potassium, chloride, urea, creatinine, glucose, haemoglobin A_1c_, calcium, magnesium, phosphate, uric acid, total protein, albumin, total bilirubin, alanine transaminase, alkaline phosphatase, gamma glutamyltransferase, creatine kinase, lipase, total cholesterol, triglycerides, high-density lipoprotein cholesterol, haptoglobin, complement 3 (C3), complement 4 (C4), immunoglobulin G, immunoglobulin A, immunoglobulin M, iron, and transferrin. Serum kappa and lambda free light chains were measured with Freelite^®^ assays (The Binding Site Group Ltd^©^, Birmingham, United Kingdom) on cobas^®^ c501. Sixteen analytes were measured on cobas^®^ e601 (Roche Diagnostics GmbH, Mannheim, Germany) analysers: ferritin, vitamin B12, serum folate, prostate specific antigen, thyroid stimulating hormone, free thyroxine, free triiodothyronine, cortisol, follicle stimulating hormone, luteinising hormone, testosterone, sex hormone binding globulin, insulin, prolactin, parathyroid hormone, and 25-hydroxy vitamin D. Renin, aldosterone, insulin-like growth factor 1, and C-peptide were measured on the DiaSorin Liaison^®^ XL (DiaSorin, Saluggia, Italy) analyser. Most analytes were tested on serum, except for parathyroid hormone and renin which were tested on K_2_EDTA plasma, glucose tested on NaFl/KOx plasma, and haemoglobin A_1c_ tested on K_2_EDTA whole blood.

The analytical coefficient of variation for each analyte was determined from 1 year’s quality control data and have been provided to demonstrate performance, and the potential contribution of coefficient of variation to total error in this study.

### Statistical analysis

Technical validation data were captured and analysed in Microsoft^©^ Excel^®^ (Microsoft Corporation, Redmond, Washington, United States). The sigma metric was used as a performance metric to assess the technical quality of blood collection tubes, by calculating the defect rate across all 11 QIs. Each QI per tube was treated as a defect opportunity. Total observed defects were converted into defects per million opportunities using an online calculator.^16^ To adhere to typical performance standards observed in industry processes, a minimum sigma metric of 4 (6210 defects per million opportunities) was deemed to be the acceptable minimum threshold for the candidate tube.^16^ Additionally, the sigma metric of the candidate tube was required to be equal to or greater than the sigma metric of the comparative tube.

Clinical validation data were analysed using EP Evaluator^TM^ (Data Innovations LLC, South Burlington, Vermont, United States) and Excel^®^. Passing-Bablok regression was used to assess correlation, with acceptability based on the 95% confidence interval (CI) for the slope including 1 (no proportional difference) and the intercept including 0 (no systematic difference).^17^ Given the anticipated strong correlation between comparisons of the same analyte, the correlation coefficient (*R*) was likely to be highly influenced by the range of measurement results; *R* > 0.95 was deemed acceptable.^18^ Bland-Altman plots were used to assess differences between the tubes, with normality evaluated via histograms in EP Evaluator. For analytes with non-normal distribution, pairwise (Walsh) averages of the differences were calculated in a trial version of Minitab^®^ (Minitab, LCC, State College, Pennsylvania, United States) with subsequent determination of the Hodges-Lehmann point estimator (median) and Tukey two-sided CIs.^15^

Average percentage bias was deemed acceptable if it was at or below the desirable bias calculated from the EFLM Biological Variation Database^19^ or Ricos criteria.^20^ For analytes tested at two laboratories, bias exceeding the desirable bias was acceptable if the 95% CI included the bias specification.^15^ Analytes that failed both Passing-Bablok regression and Bland-Altman analysis underwent total error calculation (% total error = % bias + 1.65*CVa), compared against EFLM Biological Variation Database or Ricos total allowable error. If the % total error exceeded the total allowable error, the clinical significance of the differences rendered the tube unsuitable for the intended purpose. Outlier detection used the ‘Extreme Studentized Deviate’ method in EP Evaluator.^15^

## Results

### Technical validation

During the pre-check, no major physical defects were observed in any candidate or comparative tubes. All candidate tubes were ‘Conformité Européenne’ marked, with expiry dates, lot numbers, and clear fill lines. No tubes were excluded during the pre-check.

The primary reasons for exclusion in assessing the remaining technical QIs were primarily due to missing data and underfilling caused by difficult phlebotomy. No haemolysed samples were attributed to phlebotomy difficulties (Table 2 and Table 3).

**TABLE 2:** The number of defects observed and defect opportunities for 11 quality indicators assessed during technical validation – Cape Town, South Africa, October 2021.

Variable	SS				K_2_EDTA				LiH				NaFl/KOx			
	BD		CT		BD		CT		BD		CT		BD		CT	
	Observed defects	Defect opportunities	Observed defects	Defect opportunities	Observed defects	Defect opportunities	Observed defects	Defect opportunities	Observed defects	Defect opportunities	Observed defects	Defect opportunities	Observed defects	Defect opportunities	Observed defects	Defect opportunities
**VACUCARE versus BD**																
Manufacturing defects	0	75	0	75	0	75	0	75	0	25	0	25	0	25	0	25
Did not properly fit into the blood collection device	0	50	0	50	0	75	0	75	0	25	0	25	0	25	0	25
Failed to form a vacuum	0	50	0	50	0	75	0	75	0	25	0	25	0	25	0	25
Cap leakage (specifically where the rubber was pierced)	0	50	0	50	0	75	0	75	0	25	0	25	0	25	0	25
External surface contamination after venepuncture	0	50	0	50	0	75	0	75	0	25	0	25	0	25	0	25
Undue clotting in K_2_EDTA tubes	n/a	n/a	n/a	n/a	0	50	0	50	n/a	n/a	n/a	n/a	n/a	n/a	n/a	n/a
Incomplete clotting in serum tubes	0	49†	0	49†	n/a	n/a	n/a	n/a	n/a	n/a	n/a	n/a	n/a	n/a	n/a	n/a
Broke or spilled blood during centrifugation	0	49†	0	49†	0	25	0	25	0	24†	0	24†	0	25	0	25
Underfilling	9	48†,‡	0	49†	0	75	0	75	1	24†	0	24†	0	24†	0	25
Inappropriate gel position after centrifugation	0	49†	0	49†	n/a	n/a	n/a	n/a	n/a	n/a	n/a	n/a	n/a	n/a	n/a	n/a
Haemolysis	0	50	1	50	n/a	n/a	n/a	n/a	0	25	0	25	n/a	n/a	n/a	n/a
Total	9	520	1	521	525	525	0	525	1	198	0	198	0	174	0	175
efects per million opportunities	17 308	-	1 919	-	0	-	0	-	5051	-	0	-	0	-	0	-
Sigma metric	3.7	-	4.4	-	>6	-	>6	-	4.1	-	>6	-	>6	-	>6	-
**VACUETTE^®^ versus BD**																
Manufacturing defects	0	75	0	75	0	75	0	75	0	25	0	25	0	25	0	25
Did not properly fit into the blood collection device	0	50	0	50	0	75	0	75	0	25	0	25	0	25	0	25
Failed to form a vacuum	0	50	0	50	0	75	0	75	0	25	0	25	0	25	0	25
Cap leakage (specifically where the rubber was pierced)	0	50	7	50	0	75	18	75	0	25	2	25	0	25	6	25
External surface contamination after venepuncture	0	50	0	50	0	75	0	75	0	25	0	25	0	25	0	25
Undue clotting in K_2_EDTA tubes	n/a	n/a	n/a	n/a	0	50	0	50	n/a	n/a	n/a	n/a	n/a	n/a	n/a	n/a
Incomplete clotting in serum tubes	0	50	0	49†	n/a	n/a	n/a	n/a	n/a	n/a	n/a	n/a	n/a	n/a	n/a	n/a
Broke or spilled blood during centrifugation	0	50	0	49†	0	25	0	25	0	25	0	25	0	25	0	24†
Underfilling	1	50	1	49†	0	75	7	75	0	25	0	25	0	25	0	24†
Inappropriate gel position after centrifugation	0	50	0	49†	n/a	n/a	n/a	n/a	n/a	n/a	n/a	n/a	n/a	n/a	n/a	n/a
Haemolysis	0	50	0	50	n/a	n/a	n/a	n/a	1	25	0	25	n/a	n/a	n/a	n/a
Total	1	525	8	521	0	525	25	525	1	200	2	200	0	175	6	173
Defects per million opportunities	1905	-	15 355	-	0	-	47 619	-	5000	-	10 000	-	0	-	34 682	-
Sigma metric	4.4	-	3.7	-	>6	-	3.2	-	4.1	-	3.9	-	>6	-	3.4	-

BD, BD Vacutainer^®^; SS, serum tube with gel separator; K_2_EDTA, dipotassium ethylenediaminetetraacetic acid; LiH, lithium heparin; NaFl/KOx, sodium fluoride/potassium oxalate; CT, candidate tube.

† , excluded due to missing centrifugation data (*n* = 1);

‡ , excluded due to difficult phlebotomy (*n* = 1).

VACUCARE tubes achieved sigma metrics of 4 or higher across all comparisons. In contrast, BD Vacutainer^®^ SS had sigma metrics below 4 in three of four comparisons (BD vs. VACUCARE, VACUTEST^®^, and V-TUBE^™^), mainly due to underfilled tubes in the latter two comparisons. VACUETTE^®^ tubes also fell below a sigma metric of 4, primarily due to cap leakage after needle withdrawal. For VACUTEST SS and lithium heparin tubes, haemolysis and underfilling contributed to their sigma metrics falling below 4.

The Modular Pre-analytics system could not uncap VACUETTE^®^ tubes without hardware modifications, which were unavailable because of the manufacturer’s discontinuation of the Modular Pre-analytics system. Similarly, the *Probenverteiltechnik* cobas® p 612 pre-analytical system could not uncap V-TUBE^™^ tubes, and no application settings were available for achieving compatibility.

Phlebotomists reported usability concerns, noting that the rigid rubber in VACUTEST^®^ caps made tubes difficult to puncture and detach from collection devices, often causing needle displacement during the procedure. VACUETTE^®^ tube caps were shorter in length compared to BD Vacutainer^®^, resulting in increased angular movement within the collection device. In contrast, VACUCARE and V-TUBE^™^ tubes were user-friendly.

Based on sigma metrics, pre-analytical systems compatibility, and usability, VACUETTE^®^, VACUTEST^®^ and V-TUBE^™^ were disqualified.

### Clinical validation

The clinical validation results are detailed in the Online Supplementary Table 1, Online Supplementary Table 2, Online Supplementary Table 3, and Online Supplementary Table 4. The study aimed to include 50 or 25 sample pairs per comparison; however, the total sample size was occasionally reduced because of missing results or samples. Additionally, outliers were identified and excluded using EP Evaluator. For V-TUBE^™^, 25 of 50 C3 pairs tested at Groote Schuur Hospital were excluded due to a significant positive shift in internal quality control during the run.

**TABLE 3:** The number of defects observed and defect opportunities for 11 quality indicators assessed during technical validation – Cape Town, South Africa, November 2021.

Variable	SS				K_2_EDTA				LiH				NaFl/KOx			
	BD		CT		BD		CT		BD		CT		BD		CT	
	Observed defects	Defect opportunities	Observed defects	Defect opportunities	Observed defects	Defect opportunities	Observed defects	Defect opportunities	Observed defects	Defect opportunities	Observed defects	Defect opportunities	Observed defects	Defect opportunities	Observed defects	Defect opportunities
**VACUTEST** ^®^ **versus BD**																
Manufacturing defects	0	100	0	100	0	100	0	100	0	25	0	25	0	25	0	25
Did not properly fit into the blood collection device	0	73†	0	73†	0	97†,§	0	97†,§	0	23†	0	23	0	25	0	25
Failed to form a vacuum	0	73†	0	73†	0	97†,§	0	97†,§	0	23†	0	23	0	25	0	25
Cap leakage (specifically where the rubber was pierced)	0	73†	0	73†	1	97†,§	1	97†,§	0	23†	0	23	0	25	0	25
External surface contamination after venepuncture	0	73†	0	73†	0	97†,§	0	97†,§	0	23†	0	23	0	25	0	25
Undue clotting in K_2_EDTA tubes	n/a	n/a	n/a	n/a	0	50	0	50	n/a	n/a	n/a	n/a	n/a	n/a	n/a	n/a
Incomplete clotting in serum tubes	0	50	0	50	n/a	n/a	n/a	n/a	n/a	n/a	n/a	n/a	n/a	n/a	n/a	n/a
Broke or spilled blood during centrifugation	0	50	0	50	0	25	0	25	0	25	0	25	0	25	0	25
Underfilling	21	47†,‡	8	47†,‡	5	71†,‡,§	4	71†,‡,§	1	22†,‡	2	22	0	25	0	25
Inappropriate gel position after centrifugation	0	50	0	50	n/a	n/a	n/a	n/a	n/a	n/a	n/a	n/a	n/a	n/a	n/a	n/a
Haemolysis	0	50	3	50	n/a	n/a	n/a	n/a	0	25	1	25	n/a	n/a	n/a	n/a
Total	21	639	11	639	6	634	5	634	1	189	3	189	0	175	0	175
Defects per million opportunities	32 864	-	17 214	-	9464	-	7886	-	5291	-	15 873	-	0	-	0	-
Sigma metric	3.4	-	3.7	-	3.9	-	4	-	4.1	-	3.7	-	>6	-	>6	-
**V-TUBE™ versus BD**																
Manufacturing defects	0	100	0	100	0	100	0	100	0	25	0	25	-	-	-	-
Did not properly fit into the blood collection device	0	75	0	75	0	100	0	100	0	25	0	25	-	-	-	-
Failed to form a vacuum	0	75	0	75	0	100	0	100	0	25	1	25	-	-	-	-
Cap leakage (specifically where the rubber was pierced)	0	75	0	75	0	100	0	100	0	25	0	25	-	-	-	-
External surface contamination after venepuncture	0	75	0	75	0	100	0	100	0	25	0	25	-	-	-	-
Undue clotting in K_2_EDTA tubes	n/a	n/a	n/a	n/a	0	50	0	50	n/a	n/a	n/a	n/a	-	-	-	-
Incomplete clotting in serum tubes	0	50	0	50	n/a	n/a	n/a	n/a	n/a	n/a	n/a	n/a	-	-	-	-
Broke or spilled blood during centrifugation	0	50	0	50	0	25	0	25	0	25	0	25	-	-	-	-
Underfilling	20	49‡	0	50	7	74‡	0	74‡	3	24‡	1	25	-	-	-	-
Inappropriate gel position after centrifugation	0	50	0	50	n/a	n/a	n/a	n/a	n/a	n/a	n/a	n/a	-	-	-	-
Haemolysis	0	50	0	50	n/a	n/a	n/a	n/a	0	25	0	25	-	-	-	-
Total	20	649	0	650	7	649	0	649	3	199	2	200	-	-	-	-
Defects per million opportunities	30 817	-	0	-	10 786	-	0	-	15 075	-	10 000	-	-	-	-	-
Sigma metric	3.4	-	>6	-	3.8	-	>6	-	3.7	-	3.9	-	-	-	-	-

Note: V-TUBE™ versus BD (NaFl/KOx) was not evaluated.

BD, BD Vacutainer^®^; SS, serum tube with gel separator; K_2_EDTA, dipotassium ethylenediaminetetraacetic acid; LiH, lithium heparin; NaFl/KOx, sodium fluoride/potassium oxalate; CT, candidate tube.

† , excluded due to missing phlebotomy data (*n* = 2);

‡ , excluded due to difficult phlebotomy (*n* = 1);

§ , not drawn due to difficult phlebotomy (*n* = 1);

¶, not evaluated.

Most analytes had correlation coefficients above 0.95, except sodium, potassium, and magnesium, which consistently fell below 0.95 across all comparisons. Calcium, total protein, and albumin displayed coefficients below 0.95 in most comparisons, except with VACUTEST^®^.

Passing-Bablok regression and Bland-Altman analysis indicated acceptable performance for all analytes except for aldosterone on V-TUBE^™^. Passing-Bablok regression for aldosterone on V-TUBE^™^ showed a slope of 0.927 (95% CI: 0.874, 0.961) and intercept of −13.18 (95% CI: −25.170, –6.060). The mean bias was −15.63 (95% CI: −19.70, −11.55), with a total error of 30%, below the EFLM Biological Variation Database limit of 42.8%, deeming the difference clinically insignificant.

## Discussion

In response to an impending BD Vacutainer^®^ tube shortage, technical and clinical validations were conducted to evaluate alternative tubes. A modified approach reduced the number of tubes required while maintaining rigorous standards. A key feature was the application of sigma metrics to assess technical QIs, offering an objective measure of their quality. The technical validation proved critical, as all tubes met standards in the clinical validation phase.

Sigma metrics are often used to monitor QIs in the extra-analytical phases, with a Six Sigma level corresponding to a defect rate of 3.4 defects per million opportunities. While sigma metrics often evaluate the process quality, they can also be used to assess product performance. By treating each QI per tube as a defect opportunity, a sigma metric for each blood collection tube was determined. This approach also addressed the global shortage of blood collection tubes, which prevented inclusion of the full 120-tube sample size recommended in the EFLM opinion paper.^5^

Few publications address the technical validation of blood collection tubes. To the authors’ knowledge, this is the first study to compare VACUTEST^™^ and BD Vacutainer^®^ tubes. During technical validation, VACUTEST SS failed to meet the minimum sigma metric of 4 due to underfilling and haemolysis but outperformed BD Vacutainer^®^ SS tubes overall. Notably, underfilled BD Vacutainer^®^ SS tubes were traced to a single lot (1151158), identified through reference numbers assigned during the pre-check process. The EFLM opinion paper recommends calculating percentage differences between candidate and comparative tubes for each QI, but poorly performing comparative tubes may distort results.^5^ This was similarly noted in a 2019 Turkish study, where 4 candidate tubes and 16 comparative tubes (BD Vacutainer SS) were found to be underfilled.^21^ Using sigma metrics with a pre-set acceptance threshold allows independent evaluation of candidate tubes, reducing reliance on comparative tube performance.

To the best of the authors’ knowledge, this study provides the first evaluation of VACUCARE tubes compared to BD Vacutainer^®^. However, a 2009 Korean study assessed tubes from Zhejiang GongDong Medical Technology Co., Ltd., likely using similar manufacturing technology across all their blood collection tubes.^22^ However, that study did not incorporate a technical evaluation, and it relied on Clinical Laboratory Improvement Amendments of 1988 limits instead of biological variation data, finding no significant differences between the tubes.

VACUETTE^®^ and BD Vacutainer^®^ serum tubes were compared in a 2011 Brazilian study by Lima-Oliveira et al. using a Roche cobas^®^ c501.^23^ Like the current study, clinical validation acceptance criteria were based on desirable bias, derived from biological variation. While the previous study found a clinically significant mean difference of 4.9% for magnesium, the current study demonstrated a mean difference of -0.69% (95% CI: -1.64, 0.25), well below the desirable bias of 1.62%.

A 2014 study by Bowen et. al. in the United States assessed VACUETTE^®^ and BD Vacutainer^®^ serum tubes against glass tubes lacking clot activators, separator gels, and surfactants that could potentially impact results.^24^ Both VACUETTE^®^ and BD Vacutainer^®^ tubes showed no statistically or clinically significant differences in cortisol results using the Siemens Immulite^™^ 1000 analyser. The current study found a mean difference of 1.90% (95% CI: -2.00, 5.90) for cortisol, in a direct comparison between VACUETTE^®^ and BD Vacutainer^®^ serum tubes, further supporting comparable performance.

Two Korean studies (2013, 2018) performed comparisons between BD Vacutainer^®^ and V-TUBE^™^ tubes for routine analytes in healthy volunteers and patients. One study showed statistically significant differences for sodium, chloride, alkaline phosphatase, high-density lipoprotein cholesterol, whereas the other study showed differences for creatinine, high-density lipoprotein cholesterol, iron, lipase, magnesium, potassium, total cholesterol, and uric acid.^25,26^ Yet, these findings were not deemed clinically significant in the context of Clinical Laboratory Improvement Amendments of 1988 limits.

In the current study, most analytes showed strong correlation coefficients (*R* > 0.95), but sodium, potassium, and magnesium consistently fell below this threshold. Similarly, calcium, total protein, and albumin displayed correlation coefficients below 0.95 in all comparisons, except for VACUTEST^®^. Poor correlation does not necessarily indicate poor agreement between tubes, but may reflect the limited range of results in the healthy study population.^18,27^

While combining blood collection tubes and devices from different manufacturers is discouraged,^5^ the unique study circumstances required using BD devices for all candidate tubes. Sigma metrics for VACUETTE^®^ tubes consistently fell below 4 because of cap leakage from the puncture sites after needle withdrawal, posing safety concerns. However, this issue did not affect the clinical validation. Given these findings, it is advised against combining BD devices with VACUETTE^®^ tubes.

### Strengths

The strength of this validation study lies in its comprehensive assessment of analytes using VACUCARE, VACUETTE^®^, VACUTEST^®^, and V-TUBE^™^ compared to BD Vacutainer^®^ tubes. It is among the few studies reporting on the technical validation of blood collection tubes and introduces a novel assessment approach. Furthermore, it demonstrates no clinically significant differences in specialised tests between comparative and candidate tubes, using desirable bias specified by the EFLM Biological Variation Database or Ricos as acceptance criteria.

### Limitations

A key limitation was the inclusion of only healthy adult volunteers, which excluded several analytes due to low levels in this population and resulted in a narrow testing range for several analytes.

### Conclusion

The technical validation component, combined with pre-analytical system compatibility considerations, was essential and led to the exclusion of certain candidate tubes. Comprehensive technical validation should precede the clinical validation.

VACUCARE, VACUETTE^®^, VACUTEST^®^ and V-TUBE^™^ are viable alternatives to BD Vacutainer^®^. However, based on technical validation results, VACUCARE was identified as the most suitable interim replacement for BD Vacutainer^®^ during the shortage.
